# An immunohistochemical study of endocrine cells in the digestive tract of *Varanus salvator* (Reptile: Varanidae)

**DOI:** 10.14202/vetworld.2020.1737-1742

**Published:** 2020-09-01

**Authors:** Mahfud Mahfud, Ernawati Ernawati, Nur R. Adawiyah Mahmud, Teguh Budipitojo, Hery Wijayanto

**Affiliations:** 1 Department of Biology Education, University of Muhammadiyah Kupang, East of Nusa Tenggara Province, Indonesia; 2 Department of Veterinary Anatomy, Faculty of Veterinary Medicine, Gajah Mada University, Yogyakarta, Indonesia

**Keywords:** digestive tract, endocrine cell, immunohistochemistry, *Varanus salvator*

## Abstract

**Aim:**

The aim of the study was to identify the distribution pattern and frequency of endocrine cell types in the digestive tract of *Varanus salvator*.

**Materials and Methods:**

The presence of endocrine cells (glucagon, somatostatin, and serotonin) in the digestive tract (esophagus, stomach, and intestine) was detected using the avidin-biotin complex (ABC) method.

**Results:**

Three types of endocrine cells immunoreactive to antisera glucagon, serotonin, and somatostatin were found in the caudal portion of the small and large intestines but were not observed in the esophagus, stomach, and caput and medial sections of the small intestine. Endocrine cells distributed in the digestive tract of *V. salvator* vary in color intensity, from weak to sharp, in response to the primer antibody.

**Conclusion:**

Endocrine cells in the digestive tract that is immunoreactive to glucagon, somatostatin, and serotonin are those found in the caudal portion of the small and large intestines. They are varied in distribution pattern, frequency, and color intensity.

## Introduction

The water monitor (*Varanus salvator*) (Family: Varanidae) [1] is found throughout South and Southeast Asia [2], as well as Indonesia, including East Nusa Tenggara, notably on the beach forest and mangrove habitats of Flores and Alor Islands [3]. *V. salvator* has been categorized as “least concern” on the ICUN red list of threatened species [4] and is documented in Appendix II of the CITES-listed species. This species is still hunted for commercial purposes. Its skin and meat are traded as raw materials for medicine, food, clothing, and for use in the domestic industry [5]. If this hunting and trading continue, it will cause an imbalance in the beach forest and mangrove ecosystem, leading to a decrease in the water monitor population. To prevent the species from becoming endangered, breeding efforts must be made. Comprehensive biological information for the water monitor is needed to support the breeding and raising of this animal. One of the most important elements to consider is the *V. salvator*’s digestive tract. This information is important in helping correlate the adaptation type and dietary habits of *V. salvator* before breeding. A previous study reported that the water monitor’s digestive tract consists of the esophagus, stomach, small intestine (*intestinum tenue*), large intestine (*intestinum crissum*), and cloaca. The water monitor’s stomach differed from other animals with single-chambered stomachs, which commonly have a major and minor curvature. Its striated stomach was the biggest organ in the digestive tract [6]. The cecum was not found in its intestine, and there were goblet cells between epithelial cells of the intestine, with different cell shapes in several parts of the intestine [7].

All bodily activities are controlled and coordinated by the nervous and endocrine systems. The digestive system is considered the largest organ of the endocrine system in an animal’s body. Endocrine organs release hormones that help them to regulate their own primary activity. These hormones are chemicals produced by the wall of the digestive tract that stimulates or inhibits target tissues in the gastrointestinal tract or related organ [8]. Reptiles are ­cold-blooded vertebrates living and reproducing under conditions dictated by the land environment. The warmer Mesozoic Era was favorable to the reptile’s development and diversification, but the colder modern earth has imposed severe limitations on the land-living and breeding of cold-blooded vertebrates [9]. These conditions also influence the reptile’s eating behavior, resulting in them clawing and pouncing on their prey. However, the reptile’s feeding response is very good, which can be influenced by the regulation of digestive physiology [10].

Several animal studies on the distribution patterns of endocrine cells in the digestive tract were reported, including *Tragulus javanicus* [11], *Babyrousa ­babyrussa* [12], *Manis javanica* [13], *Muntiacus muntjak* [14], *Rhinella* *icterica* [15], and *Hystrix javanica* [16]. Regarding reptiles, almost all previous studies were reported on small reptiles such as *Gekko japonicus*, *Eumeces chinensis*, *Sphenomorphus indicus*, *Eumeces elegans* [17], and *Tropidurus torquatus* [18]. The patterns, activities, and dietary habits of mammals and reptiles are different and thus will affect the morphology and distribution of their endocrine cells in the digestive tract.

The aim of the study was to identify the distribution patterns and frequency of endocrine cells in the digestive tracts of *V. salvator* as this data has not thus far been reported.

## Materials and Methods

### Ethical approval

The present study was approved by the Ethics Committee for Animal of Faculty of Veterinary Medicine IPB Unity (No. 002/KEH/SKE/VI/2014).

### Study period and location

This study was conducted in the Microanatomy Laboratory, Faculty of Veterinary Medicine, University of Gadjah Mada, Yogyakarta, Indonesia, from June to July 2019.

### Collecting and preparing the biological material

The present study used the digestive tract of the same young adult *V. salvator* that was obtained in a previous study [6,19]. The sample was anesthetized with a combination of ketamine (50 mg/kg) and xylazine (10 mg/kg) intramuscularly in the thigh muscle. After anesthetization, an incision was made in the median part of the body from the perineum to sternum. A portion of the breastbone was cut to provide access to the heart. Exsanguination was performed by making an incision in the right atrium of the heart and pushing the cannula of 0.9% sodium chloride into the left ventricle to irrigate until the liquid draining from the right atrium appears clear. Then, the fixation procedure was performed using 4% paraformaldehyde perfused to the beating heart. The fixative liquid was injected several times to the hollow organs to maximize fixation. The organs were immersed in 4% paraformaldehyde for 2-3 days, and then placed in 70% alcohol to be stored for future use.

**Figure-1:**
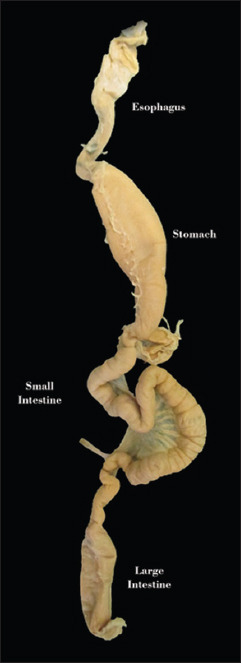
Anatomy of the digestive tract of *Varanus salvator*.

The two male water monitors were collected from Bogor Regency, Indonesia, with 4.560±6.505 cm snout-vent length. The animals were anesthetized with a combination of ketamine 50 mg/kg body weight and xylazine 10 mg/kg body weight intramuscularly at the thigh muscle. The digestive tracts of the animals were separated and used as samples in this study. Only one digestive tract from one sample animal was used for further study in this research. The sampling portions are shown in Figure-1. Tissue samples were fixed in 4% paraformaldehyde, dehydrated through an ethanol-xylene series, and embedded in paraffin. Sections were cut at 5 μm thickness and mounted on gelatin-coated glass slides.

### Immunohistochemical study

The sections were stained immunohistochemically using the avidin-biotin complex (ABC) method [20]. The sections were dewaxed and rehydrated according to the routine protocol. They were incubated in aquadest and placed in a microwave oven for 10 min for antigen retrieval. Subsequently, the sections were incubated in 3% H_2_O_2_ solution in methanol for 30 min to block any endogen peroxidase activity. Next, they were incubated with blocking serum for 1 h in a humid chamber at room temperature.

The esophagus, stomach, and intestine sections were first incubated overnight at 4°C in the respective primary antibodies (Table-1). The negative controls were processed by replacing the primer antibody with phosphate-buffered saline (PBS). Next, the sections were incubated in Poly-HRP Rabbit Anti-Goat as a secondary primer for 1 h in a humid chamber at room temperature. Subsequently, the sections were washed in PBS 3 times for 3 min, and finally immersed in 3,3-diaminobenzidine (DAB) for 5-30 min in darkroom to develop the immunoreactivity. After washing in distilled water, the sections were slightly counterstained with Harris hematoxylin, dehydrated, cleared in xylene, and mounted using Entellan (MilliporeSigma, Burlington, MA).

**Table-1:** Details of primary antibodies used in this study.

Primary antibody	Code number	Working dilution	Source
Glucagon	RPN1602	1:2000	Amersham International plc., Amersham, UK
Somatostatin	Lot27092	1:5000	Immuno Nuclear Corp., Stillwater, MN, USA
Serotonin	Sero-2-3	1:5000	J. Nishitsutsuji-Uwo, Shionogi Co., Kyoto, Japan

### Observation and photomicrography

The sections were examined with a light microscope (Nikon YS100, Tokyo, Japan). Photographs were taken with the Optilab Advance Plus (Wyatt Technology, Santa Barbara, CA). Only immunoreactive endocrine cells with discernible nuclei were considered. The relative frequencies of endocrine cells were graded subjectively into five groups: (–), absent; (+), few and not detected in every section; (++), few; (+++), moderate; and (++++), numerous.

## Results

This study found that the three types of endocrine cells were detected only in the intestine of the *V. salvator* digestive tract, while not found in the esophagus and stomach. Glucagon-, somatostatin- and serotonin-IR cells were identified predominantly in the large intestine of the *V. salvator*. Somatostatin- and serotonin-IR cells were also identified in the caudal section of the *V. salvator*’s small intestine, but ­glucagon-IR cells were not found here. The distribution patterns and frequencies of the endocrine cells in the digestive tract of the *V. salvator* are shown in Table-2.

**Table-2:** Distribution and frequency of the endocrine cells of the digestive tract of *Varanus salvator.*

Antibodies	Esophagus	Stomach		Small intestine			Large intestine
	
		Fundus	Pylorus	Caput	Medial	Caudal	
Glucagon	−	−	−	−	−	−	++++
Somatostatin	−	−	−	−	−	++	++++
Serotonin	−	−	−	−	−	+	+++

−=Absent, +=Few and not detected in every section, ++=Rare in number, +++=Moderate, ++++=Numerous

### Glucagon-IR cells

**Figure-2:**
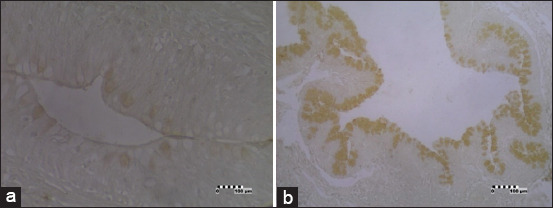
Photomicrographs of glucagon-immunoreactive (IR) in the large intestine of *Varanus* *salvator*. Glucagon-IR cells between the epithelial cells (brown) (×10). (a) Proximal and (b) distal parts of large intestine. Bar: 100 µm.

Glucagon-IR cells were found only in the large intestine of the *V. salvator* digestive tracts (Table-2). The endocrine cells were spread broadly on the apical surface of the epithelia of the large intestines (Figure-2). This cell type showed the open-typed endocrine cells. The frequency of glucagon-IR cells was numerous throughout the epithelial wall, causing the epithelial cells to be closed. Although the distribution patterns and frequencies of glucagon-IR cells were numerous throughout the epithelial cells, endocrine cells in the large intestine showed different color intensity between the proximal and distal parts of the large intestine. The endocrine cells in the proximal part of the large intestine showed weak color intensity, while the distal part showed sharp color intensity toward the glucagon primary antibody.

### Somatostatin-IR cells

**Figure-3:**
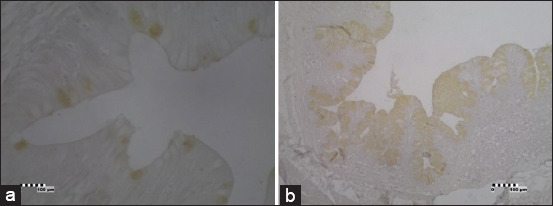
Photomicrographs of somatostatin-immunoreactive (IR) in the intestine of *Varanus* *salvator*. (a) Small intestine (caudal), somatostatin-IR cells between the epithelial cells (brown) (×40); (b) Large intestine, somatostatin-IR cells between the epithelial cells (brown) (×10). Bar: 100 µm.

Somatostatin-IR cells were also classified as open-typed endocrine cells and were found predominantly throughout the apical surface of the epithelia of the large intestines (Figure-3). This cell type was also found in the caudal section of the small intestine of the *V*. *salvator*. Somatostatin-IR cells were not found in the esophagus and stomach. The endocrine cells in the large intestine showed different color intensity between the proximal and distal parts of the large intestine. The endocrine cells in the proximal part of large intestine showed weak color intensity, while the distal part showed sharp color intensity toward the somatostatin primary antibody.

### Serotonin-IR cells

**Figure-4:**
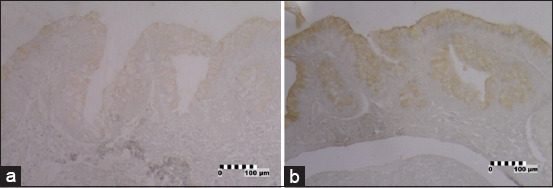
Photomicrographs of serotonin-immunoreactive (IR) in the intestine of *Varanus* *salvator*. (a) Small intestine (caudal), serotonin-IR cells between the epithelial cells (brown) (×40); (b) Large intestine, serotonin-IR cells between the epithelial cells (brown) (×10). Bar: 100 µm.

Numerous serotonin-IR cells were found in all parts of the epithelia of large intestines and a few in the caudal section of the small intestines of the *V. salvator* (Figure-4). This endocrine cell type was also not found in the esophagus and stomach. Serotonin-IR cells found in the caudal section of the small intestine were minimal with weak color intensity, while serotonin-IR cells were found in greater number in the large intestine and showed lower color intensity.

## Discussion

All bodily activities are controlled and coordinated by the nervous and endocrine systems. The digestive system is considered the largest organ of the endocrine system in an animal’s body [8]. A previous study described that the anatomy of the *V. salvator* digestive tract consists of the esophagus, stomach, small intestine, large intestine, and cloaca [6], and the accessory digestive organs are the oral organs, liver, gallbladder, and pancreas [21]. A study on the distribution and frequency of endocrine cells in the digestive tract was focused on mammals such us *Tragulus javanicus* [11], *Bubalus bubalis* [22], *Babyrousa babyrussa* [12], *Manis javanica* [13], and *Muntiacus muntjak* [14]. Several studies that have reported on reptiles focused on small reptiles, including *Tropidurus* *torquatus* [18], *Gekko japonicus*, *Eumeces chinensis*, *Sphenomorphus* *indicus*, and *Eumeces* *elegans* [17].

Eating behavior is a complex response to various internal and external factors. The aim of this behavior is to preserve energy homeostasis, body weight stability, and to maintain health [23]. The digestive system controls eating behavior, digestion, and nutrition absorption while producing several peptides that modulate the food interest, satiety, and motility of the intestine [24].

The gastrointestinal (GI) tract contains enteroendocrine (EE) cells, which are a special epithelial cell type that release several hormones and peptides as a response to environmental cues [25]. The role of the endocrine system is to detect the components of the intestinal lumen, monitor the status of body energy, and as physiologic response to control metabolic homeostasis in the whole body post-prandial in response to food consumed. Collectively, the GI endocrine system produces more than 20 different hormones that mediate the effect through neuro-, auto, and paracrine mechanisms that are distributed in ten different populations of EE cells [26]. Three of the endocrine cells found in the digestive system are glucagon, somatostatin, and serotonin.

### Glucagon-IR cells

Glucagon is found not only in alpha cells in the pancreas but also outside of the pancreas (extrapancreatic) in the digestive tract. Pancreatic glucagon secretion is different than extrapancreatic glucagon secretion, which resembles other endocrine cells that stimulate luminal nutrition of the gastrointestinal tract as secretion mediators [27].

Glucagon in the gastrointestinal tract is one of the digestive hormones that trigger insulin release, acts as a satiety signal, regulates food desires, and slows gastric emptying [28] by inhibiting gastrointestinal motility [29].

Glucagon-IR cells in the digestive tract of the *V. salvator* were found only in the large intestine. This study found both similar and different distribution patterns and frequency results when compared to other reptiles. Glucagon-IR cells in the digestive tract were found only in the stomach of the *Gekko* *japonicus*; in the fundus, ileum, and rectum of the *Eumeces chinensis*; in the ileum and rectum of the *Sphenomorphus* *indicus*; but were not found in the *Eumeces elegans* [17]. Glucagon-IR cells also were not found in the intestine (small and large) of the *Tropidurus torquatus* [18].

This pattern has similarities to other mammal groups. The distribution patterns of glucagon-IR cells are varied for every species. Some are found in all parts and some only in a few parts of the digestive tract. Glucagon-IR cells were found in all parts of the digestive tract in *Suncus murinus* [30] and *Tupaia belangeri* [31] but in only a few parts in *Bubalus bubalis* [22], *Tragulus javanicus* [11], *Babyrousa babyrussa* [12], *Manis javanica* [13], and *Muntiacus muntjak* [14]. None were found in *Meriones unguiculatus* [32].

### Somatostatin-IR cells

In the digestive system, somatostatin-IR is found in D cells of the endocrine epithelium in the mucosa layer of the digestive tract, and its secretion is regulated by the autonomic nervous system and other regulatory substances in the intestine, including gastrin, cholecystokinin (CCK), and substance P. In the digestive tract, somatostatin-IR inhibits the secretion of gastric acid, gastric emptying, intestine motility, and the release of insulin, glucagon, and several kinds of digestive hormones [33], digestive enzymes, and bile. In the intestine, somatostatin-IR inhibits the secretion of intestinal hormones (gastrin, CCK, gastric inhibitory polypeptide, vasoactive intestinal peptide, enteroglucagon, and motilin) and colonic fluid [34].

Somatostatin-IR cells in the digestive tract of the *V. salvator* were found in the large intestine and in the caudal section of the small intestine but were not found in the esophagus, stomach, and caput andmedial sections of small intestine. Varied distribution of somatostatin-R cells was also found in the digestive tracts of other reptiles. Somatostatin-IR cells were detected in the stomach and duodenum of *Gekko* *japonicus*, in the stomach of *Sphenomorphus* *indicus* and *Eumeces* *elegans*, and in all parts of the digestive tract of *Eumeces* *chinensis* [17]. Somatostatin-IR cells were not detected in the intestine (small and large) of *Tropidurus torquatus* [18].

Varied distribution patterns and frequency of somatostatin-IR cells were also found in mammals. Somatostatin-IR cells were found only in the pylorus gland of *Manis* *javanica* [13] and were found in all parts of the digestive tract of *Muntiacus* *muntjak* except in the colon and rectum [14].

### Serotonin-IR cells

Most of the serotonin (5-HT) in the GI tract is released by enterochromaffin cells (EC) and from half of all the enteroendocrine cells in the body [35]. EC cells release serotonin in response to physiologic impulses involving nutrient consumption. [36]. Serotonin release has some role in regulating intestinal motility and other important responses to nutrient consumption such as glucose adsorption and fluid balance [37]. The nutrient response resulting from serotonin release from EC depends on GI location [38]; therefore, this can be affected by the difference in serotonin levels along the intestine.

In addition to somatostatin-IR cells, serotonin-IR cells were also found in the caudal section of the small intestine and in the large intestine of *V. salvator* digestive tracts. The frequency of serotonin-IR cells in the large intestine was moderate compared to the caudal section of the small intestine. These results were similar to the serotonin-IR cells in the small and large intestine of *Tropidurus torquatus* [18] and in the gastric glands of mammals, including *Manis* *javanica* [13]. Serotonin-IR cells were detected in all parts of the digestive tract of *Muntiacus muntjak* with varied frequency [14].

## Conclusion

Endocrine cells in the digestive tract that is immunoreactive to glucagon, somatostatin, and serotonin are in the large intestine and the caudal section of the small intestine of *V. salvator*. They vary in distribution pattern, frequency, and color intensity.

## Authors’ Contributions

MM: Chief of the research, contributed in organization and designed the research and manuscript, data collection, and analysis. EE: Designed the research, method, data collection, and analysis, NRAM: Data collection, analysis and manuscript drafting. TB: Directed in method and data collection. HW: Directed in method and data collection. All authors read and approved the final manuscript.

## References

[1] Böhme W (2003). Checklist of the living monitor lizards of the world (family *Varanidae*). Zool. Verh. Leiden.

[2] Koch A, Auliya M, Schmitz A, Kuch U, Böhme W (2007). Morphological studies on the systematics of South East Asian water monitors (*Varanus salvator* Complex):Nominotypic populations and taxonomic overview. Mertensiella.

[3] Shine R, Harlow P.S, Keogh J.S (1996). Commercial harvesting of giant lizards:The biology of water monitors *Varanus salvator* in Southern Sumatra. Biol. Conserv.

[4] Bennett D, Gaulke M, Pianka E.R, Somaweera R, Sweet S.S (2010). Varanus salvator. The IUCN Red List of Threatened Species 2010:e.T178214A7499172.

[5] Putra Y.A, Masy'ud B, Ulfah M (2008). Diversity of Medicinal Animals in Betung Kerihun National Park, West Kalimantan, Indonesia. Med. Konservasi.

[6] Mahfud M, Ihwan I (2016). The anatomy of the digestive tract of asian water monitor (*Varanus salvator*) (Reptile *Varanidae*) (Anatomi saluran pencernaan biawak air asia (*Varanus salvator*) (Reptile *Varanidae*)). In:Proceeding. National Seminar of Science and Technology 3^th^ October 28^th^-29^th^ 2016, PA-36-A-41. University of Nusa Cendana Kupang, Kupang.

[7] Hamny H, Mulyani S, Masyitha D, Wahyuni S, dan Jalaluddin M (2015). The Anatomical and Histological Morphology of Intestinal Water Monitor (*Varanus salvator*). J. Vet.

[8] Kardong K.V (2008). vertebrates:Comparative Anatomy, Function, Evolution.

[9] Yadav M (2008). reptilian Endocrinology.

[10] Secor S.M, Diamond J (1998). A vertebrate model of extreme physiological regulation. Nature.

[11] Agungpriyono S, Yamada J, Kitamura N, Yamamoto Y, Said N, Sigit K, Yamashita T (1994). Immunohistochemical study of the distribution of endocrine cells in the gastrointestinal tract of the lesser mouse deer (*Tragulus javanicus*). Acta Anat Basel.

[12] Agungpriyono S, Macdonald A.A, Leus K.Y.G, Kitamura N, Adnyane I.K.M, Goodall G.P, Hondo E, Yamada J (2000). Immunohistochemical study on the distribution of endocrine cells in the gastrointestinal tract of the babirusa *Babyrousa babyrussa*(Suidae). Anat. Histol. Embryol.

[13] Nisa C, Kitamura N, Sasaki M, Agungpriyono S, Choliq C, Budipitojo T, Yamada J, Sigit K (2005). Immunohistochemical study on the distribution and relative frequency of endocrine cells in the stomach of the Malayan Pangolin *Manis javanica*. Anat. Histol. Embryol.

[14] Adnyane I.K.M, Zuki A.B, Noordin M.M, Agungpriyono S (2011). Immunohistochemical study of endocrine cells in the gastrointestinal tract of the barking deer *Muntiacus muntjak*. Anat. Histol. Embryol.

[15] Machado-Santos C, Pelli-Martins A.A, Abidu-Figueiredo M, de Brito-Gitirana L (2014). Histochemical and immunohistochemical analysis of the stomach of *Rhinella icterica (Anura Bufonidae*). J. Histol.

[16] Budipitojo T, Fibrianto Y.H, Mulyani G.T (2016). The types of endocrine cells in the pancreas of Sunda porcupine (*Hystrix javanica*). Vet. World.

[17] Huang X.G, Wu X.B (2005). Immunohistochemical study on gastrointestinal endocrine cells of four reptiles. World. J. Gastroenterol.

[18] Firmiano E.M.S, Cardoso N.N, Sales A, Santos M.A.J, Mendes A.L.S, Nascimento A.A (2017). Immunohistochemical study of the six types of endocrine cells in the enteropancreatic system of the lizard *Tropidurus torquatus*(*Squamata Tropiduridae*). Eur. Zool. J.

[19] Mahfud M, Ernawati E (2017). Histochemical study of water monitor intestine (*Varanus salvator*) (Reptile *Varanidae*). (Studi histokimia usus biawak biawak air asia (*Varanus salvator*) (Reptil *Varanidae*)). In:Proceeding. National Seminar of Biology Education and Science 2^th^ September 23^th^ 2017. University of Muhammadiyah Kupang Kupang.

[20] Hsu S.M, Raine L, Fanger H.X (1981). Use of avidin-biotin peroxidase complex (ABC) in immunoperoxidase techniques:A comparison between ABC and unlabeled antibody (PAP) procedures. J. Histochem. Cytochem.

[21] Srichairat N, Taksintum W, Chumnanpuen P (2018). Gross morphological structure of digestive system in water monitor lizard *Varanus salvator*(*Squamata Varanidae*). Walailak J. Sci. Technol.

[22] Baltazar E.T, Kitamura N, Hondo E, Yamada J, Maala C.P, Simborio L.T (1998). Immunohistochemical study of endocrine cells in the gastrointestinal tract of the Philippine Carabao (*Bubalus bubalis*). Anat. Histol. Embryol.

[23] Laviano A, Di Lazzaro L, Koverech A (2018). Changes in eating behavior, taste and food preferences and the effects of gastrointestinal hormones. Clin. Nutr. Exp.

[24] Steinert R.E, Beglinger C, Langhans W (2016). Intestinal GLP-1 and satiation:From man to rodents and back. Int. J. Obes Lond.

[25] Martin A.M, Lumsden A.L, Young R.L, Jessup C.F, Spencer N.J, Keating D.J (2017a). Regional differences in nutrient-induced secretion of gut serotonin. Physiol. Rep.

[26] Mace O.J, Tehan B, Marshall F (2015). Pharmacology and physiology of gastrointestinal enteroendocrine cells. Pharmacol. Res. Perspect.

[27] Lund A, Knop F.K (2019). Extrapancreatic glucagon:Present status. Diabetes Res. Clin. Pract.

[28] Yau A.M, McLaughlin J, Maughan R.J, Gilmore W, Ashworth J.J, Evans G.H (2018). A pilot study investigating the influence of glucagon-like peptide-1 receptor single nucleotide polymorphisms on gastric emptying rate in Caucasian men. Front. Physiol.

[29] Camilleri M (2019). Gastrointestinal hormones and regulation of gastric emptying. Curr. Opin. Endocrinol. Diabetes Obes.

[30] Kitamura N, Yamada J, Watanabe T, Yamashita T (1990). An immunohistochemical study on the distribution of endocrine cells in the gastrointestinal tract of the musk shrew *Suncus murinus*. Histol. Histopathol.

[31] Yamada J, Tauchi M, Rerkamnuaychoke W, Endo H, Chungsamarnyart N, Kimura J, Kurohmaru M, Hondo E, Kitamura N, Nishida T, Hayashi Y (1999). Immunohistochemical survey of the gut endocrine cells in the common tree shrew (*Tupaia belangeri*). J. Vet. Med. Sci.

[32] Lee J.H, Lee H.S, Ku S.K, Park K.D, Kim K.S (2000). Immunohistochemical study of the gastrointestinal endocrine cells in the Mongolian gerbil *Meriones unguiculatus*. Korean J. Vet. Res.

[33] Ando H (2016). somatostatin. In:Handbook of Hormones. Academic Press.

[34] Günther T, Tulipano G, Dournaud P, Bousquet C, Csaba Z, Kreienkamp H.J, Lupp A, Korbonits M, Castaño J.P, Wester H.J, Culler M, Melmed S, Schulz S (2018). International union of basic and clinical pharmacology. CV. somatostatin receptors:Structure, function, ligands, and new nomenclature. Pharmacol. Rev.

[35] Smith T.K, Gershon M.D (2015). Cross talk proposal:5-HT is necessary for peristalsis. J. Physiol.

[36] Zelkas L, Raghupathi R, Lumsden A.L, Martin A.M, Sun E, Spencer N.J, Young R.L, Keating D.J (2015). Serotonin-secreting enteroendocrine cells respond via diverse mechanisms to acute and chronic changes in glucose availability. Nutr. Metab.

[37] Martin A.M, Lumsden A.L, Young R.L, Jessup C.F, Spencer N.J, Keating D.J (2017b). The nutrient-sensing repertoires of mouse enterochromaffin cells differ between duodenum and colon. Neurogastroenterol. Motil.

[38] Martin A.M, Lumsden A.L, Young R.L, Jessup C.F, Spencer N.J, Keating D.J (2017c). The diverse metabolic roles of peripheral serotonin. Endocrinology.

